# Postpandemic Recovery of Case Mix Index and Risk-Adjusted Mortality in US Hospitals

**DOI:** 10.1001/jamanetworkopen.2025.43398

**Published:** 2025-11-12

**Authors:** Chris DeRienzo, David Levine, Saloni Jain, Alyssa Harris

**Affiliations:** 1 American Hospital Association, Chicago, Illinois; 2 Health Research & Educational Trust, American Hospital Association, Chicago, Illinois; 3 Vizient, Irving, Texas

## Abstract

**Importance:**

The COVID-19 pandemic disrupted long-standing trends in hospital quality and patient safety, prompting questions about whether risk-adjusted outcomes have resumed their prepandemic trajectories.

**Objective:**

To determine whether trends in risk-adjusted mortality and case mix index (CMI) among hospitalized patients in hospitals have returned to prepandemic trajectories.

**Design, Setting, and Participants:**

This retrospective cohort study was a multicenter analysis that used the Vizient Clinical Data Base, which contains encounter-level administrative and financial data from more than 1300 hospitals in the US. The study included continuously reporting hospitals and examined inpatient discharges between October 2019 and March 2024. Data were analyzed between January and May 2025.

**Main Outcomes and Measures:**

Quarterly estimates of CMI and standardized mortality ratio (SMR) (observed-to-expected ratio). Ordinary least squares (OLS) regression was used to evaluate overall linear trends, and joinpoint regression was used to identify inflection points. Statistical significance was defined as *P* < .05, with slope estimates reported alongside 95% CIs.

**Results:**

Among 715 hospitals and 7 802 606 million inpatient encounters, the mean CMI increased from 1.70 in the fourth quarter (Q4) of 2019 to 1.79 in the first quarter (Q1) of 2024 (difference, 0.09; 95% CI, 0.01 to 0.17; *P* = .02). In OLS regression, CMI showed no significant overall linear trend (*R^2^* = 0.006; *P* = .77). The mean SMR decreased from 1.00 in Q4-2019 to 0.80 in Q1-2024 (difference, –0.20; 95% CI, –0.32 to –0.08; *P* = .001), with a significant linear decline across the study period (*R*^2^ = 0.735; *P* < .001). Joinpoint regression identified a CMI inflection point in Q4-2020 (slope, 1.85; 95% CI, 0.73 to 4.14; *P* < .001 before; slope, –0.30; 95% CI, –0.62 to –0.09; *P* = .006 after) and an SMR inflection point in Q3-2021, after which SMR declined significantly by –3.17% per quarter (*P* < .05).

**Conclusions and Relevance:**

In this cohort study of 715 US hospitals from 2019 to 2024, risk-adjusted in-hospital mortality declined significantly following the COVID-19 pandemic, resuming its prepandemic trajectory of improvement, while patient acuity as measured by CMI remained elevated. These findings suggest a new postpandemic baseline for patient acuity, whereas hospital mortality outcomes have returned to prior improvement trends.

## Key Points

**Question:**

Have patient safety indicators—specifically patient acuity as measured by case mix index and risk-adjusted in-hospital mortality—returned to their prepandemic improvement trajectories in US hospitals?

**Findings:**

In this cohort study of 715 continuously reporting US hospitals using data from the Vizient Clinical Data Base, risk-adjusted in-hospital mortality declined significantly from Q3-2021 through Q1-2024, while patient acuity as measured by case mix index remained elevated compared with prepandemic levels despite a post-2020 decline.

**Meaning:**

These findings suggest that while patient acuity may reflect a new postpandemic baseline, risk-adjusted mortality has resumed its prepandemic trajectory of improvement.

## Teaser

This cohort study examines trends in risk-adjusted mortality and case mix index among hospitalized patients and determines if they have returned to prepandemic trajectories.

## Introduction

Much has been written about the impact of the COVID-19 pandemic on measures of patient safety in hospitalized patients.^1,2,3^ The temporal effect of the pandemic is layered over a decades-long trend in patient safety improvement, predating the 1999 Institute of Medicine report *To Err is Human*, and reaching at least into the 1980s.^4,5,6^

As the world regains balance after years of pandemic-induced trend disruptors, a key question is whether patient safety measures have returned to their prepandemic improvement trajectories. One such measure is risk-adjusted mortality, which has shown decades of improvement with variability reported across condition level analyses.^7,8,9,10,11^ Reports also document improvement in outcomes among patients with COVID-19 during the pandemic.^9^The Vizient Clinical Data Base (CDB), widely used to study hospital performance, has been published for risk-adjusted outcomes at the condition-level analyses.^12,13,14,15^ The goal of this study was to evaluate whether U.S. hospitals have resumed prepandemic improvement trajectories in risk-adjusted mortality and case mix index following the disruptions caused by the COVID-19 pandemic.

## Methods

The CDB was queried for inpatient admissions at continuously reporting hospitals between October 2019 and March 2024. The CDB contains administrative and financial data for more than 1300 US hospitals, representing more than 10 million inpatient and 180 million outpatient visits annually. Data undergo routine validation and have been widely used in peer-reviewed studies of hospital performance and outcomes.^12,13,14,15^

A single risk model year (Academic Medical Center version 2023) was applied consistently across all quarters to estimate expected mortality. Case mix index (CMI), calculated from diagnosis-related group weights, measured patient acuity; higher values indicate greater complexity and resource intensity. Standardized mortality ratio (SMR), defined as observed-to-expected in-hospital mortality, was used for risk-adjusted outcomes (SMR of more than 1.0, higher-than-expected; SMR less than 1.0 was considered lower-than-expected).

This study used aggregate, deidentified secondary data and met exemption criteria under the code of federal regulations (CFR) 45 CFR 46.104; thus, institutional review board oversight and informed consent were not required. Reporting followed the Strengthening the Reporting of Observational Studies in Epidemiology (STROBE) reporting guideline for cohort studies. Data from the Vizient Clinical Data Base was used with permission of Vizient (Supplement 2).

### Statistical Analysis

To assess trends in patient acuity and the observed to expected standardized mortality ratio (SMR) for in-hospital mortality analyses were conducted at the hospital-quarter level using aggregated, risk-adjusted SMR estimates. Ordinary least squares (OLS) regression assessed linear trends and joinpoint regression (model selected by Weighted Bayesian Information Criterion) identified inflection points, allowing the number and location of joinpoints to be data-driven. Goodness-of-fit statistics and residual diagnostics supported model adequacy, and results were consistent with simpler linear models. Slope estimates were reported with 95% CIs. All statistical tests were 2-sided, with significance defined as *P* < .05. Analyses were conducted using SAS 9.4 (SAS Institute) and Joinpoint 5.3.0.0 (National Cancer Institute). Data were analyzed from January to May 2025.

## Results

A total of 715 hospitals were included, representing 4 cohorts (eAppendix in Supplement 1): 113 comprehensive academic medical centers (15.8%), 149 large specialized complex care medical centers (20.8%), 162 complex care medical centers (22.7%), and 291 community hospitals (40.7%).

### CMI

**Figure 1.**
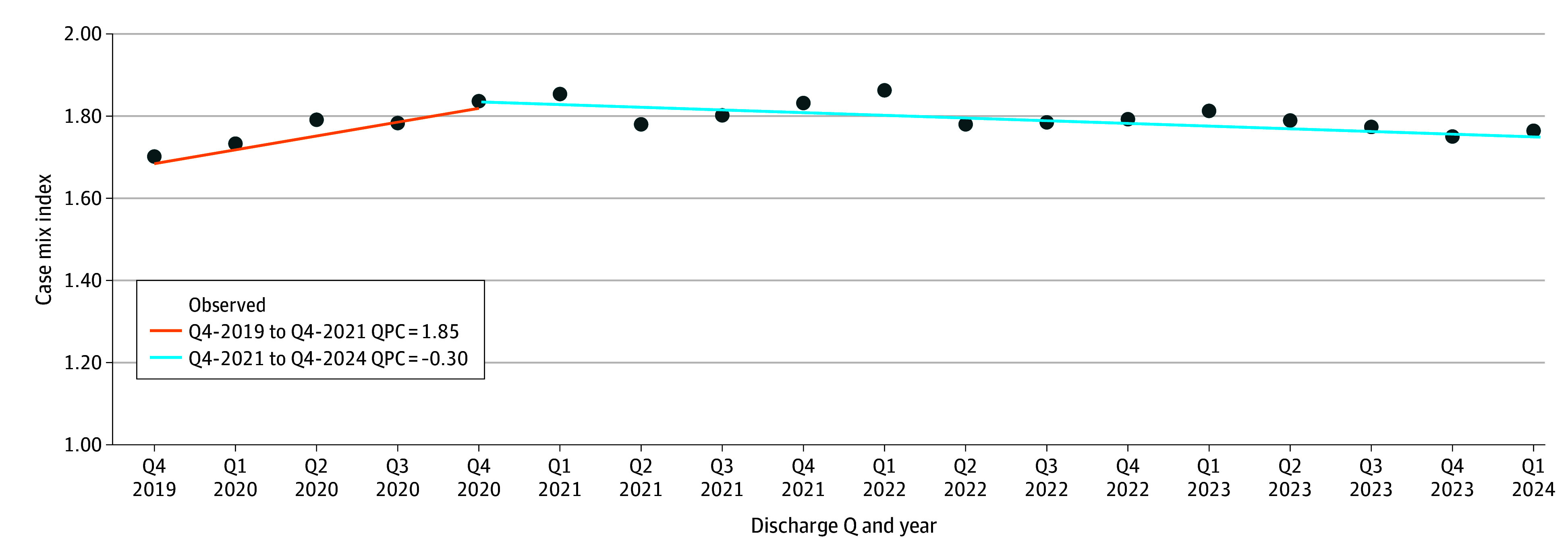
Patient Acuity Measured as Case Mix Index, October 2019 to March 2024 Q indicates quarter.

The mean CMI increased from 1.70 in the fourth quarter (Q4) of 2019 to 1.79 in the first quarter (Q1) of 2024 (difference, 0.09; 95% CI, 0.01 to 0.17; *P* = .02). OLS regression showed no overall linear trend (*R*^2^ = 0.006; *P* = .77) in CMI over the study period. Joinpoint analyses of CMI identified Q4-2020 as an infection. From Q4-2019 to Q4-2020, CMI increased (slope, 1.85; 95% CI, 0.73 to 4.14; *P* < .001), then from Q1-2021 to Q1-2024 declined (slope, −0.30; 95% CI −0.62 to −0.09; *P* = .006) (Figure 1).

### Standardized Mortality Ratio

**Figure 2.**
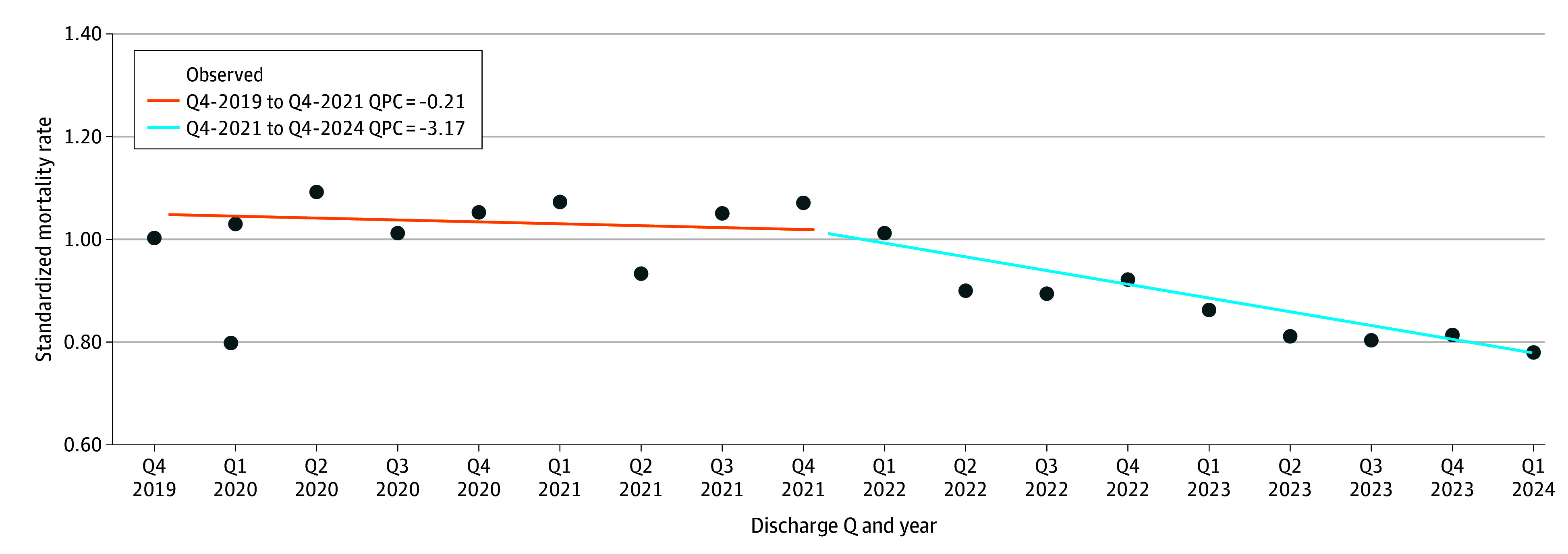
Standardized Mortality Rate Trends, October 2019 to March 2024 Q indicates quarter.

The mean SMR decreased from 1.00 in Q4-2019 to 0.80 in Q1-2024 (difference, –0.20; 95% CI, –0.32 to –0.08; *P* = .001), with a significant linear decline across the study period (*R*^2^ = 0.735; *P* < .001). Joinpoint regression identified Q3-2021 as an inflection: from Q4-2019 to Q3-2021, SMR remained stable (nonsignificant quarterly percentage change [QPC] of −0.21%; 95% CI, −0.72% to 0.31%). However, from Q4-2021 to Q1-2024 declined significantly (−3.17% per quarter; 95% CI, −4.81 to −1.52; *P* < .05) (Figure 2).

## Discussion

The COVID-19 pandemic introduced a profound and immediate disruption to hospitals, fundamentally altering patient populations, acuity distributions, and care delivery. The CDB has documented long-term improvements in hospital outcomes, and this study suggests that, while pandemic-related disruptions were substantial, trends may now be resuming their prepandemic trajectory of improvement. Prior studies report gradual national declines in hospital mortality, and the observed post-2021 quarterly SMR decrease (–3.17% per quarter) is consistent with this trajectory.

CMI rose sharply from Q4-2019 to Q4-2020, reflecting an influx of critically ill COVID-19 patients and selective admissions under capacity strain, then declined after. Although CMI in Q1-2024 remained significantly higher than in Q4-2019, the continuing downward trend suggests that acuity may be converging toward prepandemic levels. This comparison may also be partially influenced by seasonal variation in case mix, which could affect quarterly estimates.

SMR declined significantly after Q3-2021, suggesting improvements in COVID-19 management, vaccination rollouts, and system-level adaptations. The relative stability in SMR through Q2-2021 (QPC, −0.21%, not statistically significant) is reassuring given substantial disruption to care. The sustained decline in SMR suggests a return to prepandemic improvement trajectories.^15^

Risk-adjusted outcomes, such as CMI, are shaped by a decades-long trajectory of improvements in care, clinical protocols, and quality initiatives. The CMI inflection at Q4-2020 reflects pandemic-driven pressures, while the later decline may reflect an easing of pandemic-related pressures, coding shifts, or changes in hospital use. Whether this represents a stabilization to prepandemic norms or signals a fundamental shift in hospital patient composition remains unknown. SMR inflection points may also align with system adaptation and clinical milestones, including vaccines and effective therapies, such as dexamethasone and tocilizumab. While not tested here, future work could examine these associations.

Future analyses should build on these findings by incorporating risk-adjusted trends, condition-level outcomes, and evolving hospital case mix dynamics. This analysis was designed as a high-level national assessment, but heterogeneity likely exists across hospital types, regions, and patient populations. Because analyses relied on hospital-level aggregates, the models did not account for clustering of quarters within hospitals or variability across hospitals. Future work should stratify by hospital type, region, cohort, diagnostic category, and excluded sites, and apply hierarchical or mixed-effects modeling to better characterize within- and between-hospital variability. Understanding these trends will be critical in clarifying the enduring impact of the pandemic on hospital case mix and clinical outcomes. The CDB serves as a key resource for tracking hospital outcomes, including risk-adjusted mortality and acuity. The COVID-19 pandemic introduced both temporary and potentially lasting deviations from expected trajectories.

### Limitations

This study has limitations. A single, consistent risk model was applied across all quarters to minimize artifacts from changing specifications; however, this may not fully capture evolving case mix, shifts in admission thresholds, or coding changes, which could bias SMR estimates. Because quarterly hospital data are not fully independent, autocorrelation could affect variance estimates and *P* values. In addition, we did not include patient-level covariates or account for external factors such as regional COVID-19 burden, vaccination uptake, or policy changes. Sensitivity analyses excluding peak surges or stratifying by COVID vs non-COVID admissions would help clarify these effects, but they were beyond the scope of this study. Since only continuously reporting hospitals were included, results may be biased toward institutions with greater resources or stronger reporting infrastructures and may not fully generalize to all US hospitals.

## Conclusions

In this cohort study of 715 US hospitals from 2019 to 2024, risk-adjusted in-hospital mortality declined significantly following the COVID-19 pandemic, resuming its prepandemic trajectory of improvement, while patient acuity (measured by CMI) remained elevated. These findings suggested a new postpandemic baseline for patient acuity even as mortality outcomes returned to prior improvement trends.

## Notes

Supplement 1. **eMethods. **Vizient Cohort Group Descriptions **eTable.** Characteristics of hospitals included in analysis vs not included

Supplement 2.
Data Sharing Statement
