# Supplementary material for: Postpandemic Recovery of Case Mix Index and Risk-Adjusted Mortality in US Hospitals
Source: JAMA Netw Open. 2025 Nov 12;8(11):e2543398. doi: 10.1001/jamanetworkopen.2025.43398 (PMC12612936; doi:10.1001/jamanetworkopen.2025.43398)
Supplement: Supplement 1. — eMethods. Vizient Cohort Group Descriptions eTable. Characteristics of hospitals included in analysis vs not included [file jamanetwopen-e2543398-s001.pdf]

Supplemental Online Content

DeRienzo C, Levine D, Jain S, Harris A. Postpandemic recovery of case mix index and risk-adjusted mortality in US hospitals. *JAMA Netw Open*. 2025;8(11):e2543398. doi:10.1001/jamanetworkopen.2025.43398

eMethods. Vizient Cohort Group Descriptions

eTable. Characteristics of Hospitals Included in Analysis vs Not Included

This supplemental material has been provided by the authors to give readers additional information about their work.

© 2025 DeRienzo C et al. *JAMA Network Open*.

eMethods. Vizient Cohort Group Descriptions

- Comprehensive Academic Medical Centers
  - Performs a minimum of:
    - 25 solid organ transplants
    - 600 trauma cases or 1,500 acute transfers in from another acute care facility
    - 125 combined cardiothoracic and neurosurgery cases
- Large, Specialized Complex Care Medical Centers
  - Performs a minimum of:
    - 75 combined cardiothoracic and neurosurgery cases and one of the following:
    - 25 solid organ transplants
    - 600 trauma cases
    - 1,500 acute transfers in from another acute facility
- Complex Care Medical Centers
  - Hospitals not meeting the above criteria but performing between 25 and 75 combined cardiothoracic and neurosurgery cases in a year
- Community Hospitals
  - Hospitals who do not meet above criteria, excluding critical access and specialty hospitals

© 2025 DeRienzo C et al. *JAMA Network Open*.

eTable 1. Characteristics of Hospitals Included in Analysis vs Not Included

Characteristic	Hospitals in analysis<br>n (%) or Median (IQR)	Hospitals not in analysis<br>n (%) or Median (IQR)
**Vizient Cohort[a]**	n=715	n=744
Comprehensive Academic Medical Center	113 (15.8)	5 (0.7)
Large Specialized Complex Care Medical Center	149 (20.8)	31 (4.2)
Complex Care Medical Center	162 (22.7)	45 (6.0)
Community Hospital	291 (40.7)	84 (11.3)
Uncategorized [b]	0 (0)	579 (77.8)
**Rural**	95 (13.3)	273 (36.7)
**Median Bed Size, IQR**	235 (125.5, 424)	54 (25, 187)
**Census Region**		
South	232 (32.4)	282 (37.9)
Midwest	195 (27.3)	196 (26.3)
Northeast	152 (21.3)	149 (20.0)
West	136 (19.0)	117 (15.7)
**Hospital-Level Median Case Mix Index, IQR**	1.63 (1.48, 1.84)	1.46 (1.23, 1.68)

Abbreviation: IQR, interquartile range.

Note: [a] Vizient cohort definitions are provided in eMethods

[b] Hospitals must meet minimum submission volume thresholds to be assigned to a Vizient cohort group, including participation and submission of data for at least two years.

Source: Vizient Clinical Data Base, All Rights Reserved.

© 2025 DeRienzo C et al. *JAMA Network Open*.
