# Supplementary material for: Postpandemic Recovery of Case Mix Index and Risk-Adjusted Mortality in US Hospitals
Source: JAMA Netw Open. 2025 Nov 12;8(11):e2543398. doi: 10.1001/jamanetworkopen.2025.43398 (PMC12612936; doi:10.1001/jamanetworkopen.2025.43398)
Supplement: Supplement 2. — Data Sharing Statement [file jamanetwopen-e2543398-s002.pdf]

Data Sharing Statement

DeRienzo. Postpandemic Recovery of Case Mix Index and Risk-Adjusted Mortality in US Hospitals. *JAMA Netw Open*. Published November 12, 2025.
doi:10.1001/jamanetworkopen.2025.43398

Data
Data available: No

Additional Information
Explanation for why data not available: More information on Vizient data can be requested through CDPInfo@vizientinc.com
